# Cross-Sectional Study of Risky Substance Use by Injured Emergency Department Patients

**DOI:** 10.5811/westjem.2017.1.32180

**Published:** 2017-03-13

**Authors:** Valerie Strezsak, Janette Baird, Christina S. Lee, Michael J. Mello

**Affiliations:** * Brown University, Department of Emergency Medicine, Providence, Rhode Island; † Brown University, Department of Epidemiology, Providence, Rhode Island; ‡ Injury Prevention Center at Rhode Island Hospital, Providence, Rhode Island; § Northeastern University, Department of Applied Psychology, Boston, Massachusetts

## Abstract

**Introduction:**

Survey data regarding the prevalence of risky substance use in the emergency department (ED) is not consistent. The objective of this study was to identify the prevalence of risky substance use among injured ED patients based on the Alcohol, Smoking and Substance Involvement Screening Test (ASSIST v3.0). A secondary objective was to report on the feasibility of administering the ASSIST to this population, based on the time to conduct screening.

**Methods:**

This cross-sectional study used screening data from a randomized controlled trial. Injured ED patients completed the ASSIST on a tablet computer, and an ASSIST score was computed that indicated the need for a brief or intensive treatment intervention (risky use) for alcohol and other substances. For a subsample, data on time to complete each step of screening was recorded.

**Results:**

Between July 2010 and March 2013, 5,695 patients completed the ASSIST. Most (92%) reported lifetime use of at least one substance and 51% reported current risky use of at least one substance. Mean time to complete the ASSIST was 5.4 minutes and screening was considered feasible even when paused for clinical care to proceed.

**Conclusion:**

Estimates of risky substance use based on the ASSIST in our large sample of injured ED patients were higher than previously reported in other studies of ED patients, possibly due to the current focus on an injured population. In addition, it was feasible to administer the ASSIST to patients in the course of their clinical care.

## INTRODUCTION

The emergency department (ED) is an opportune setting for identifying patients with substance use problems. ED patients have higher rates of alcohol and drug use than the general population,^1^ with injured patients in particular reporting increased rates of alcohol misuse.^2^ Screening in the ED is recommended for alcohol use,^3^–^5^ and screening for other substances is an area of current research interest.^6^,^7^ Survey data regarding the prevalence of risky substance use has been assessed in two single-site studies in inner-city EDs, which found substantial differences in the prevalence of risky substance use (15%^8^ vs. 34%^9^). These differences, however, may reflect differences in the measures used to determine risky substance use rather than the rates themselves.

The Alcohol, Smoking and Substance Involvement Screening Test (ASSIST v3.0) was developed by the World Health Organization (WHO) to assess substance use in general medical settings and classify use as low, moderate, or high risk. 10 It has been used successfully to screen patients in the ED for substance use interventions.^11^,^12^ Because there are outside directives to provide screening in the ED for many substances included in the ASSIST, such as alcohol, tobacco, and opioids, demonstrating the feasibility of screening with the ASSIST in the ED setting is important to show that it can be done efficiently with a validated measure despite the difficulties inherent to screening in this setting. The objective of the current study was to describe the prevalence of substance use based on the ASSIST among injured ED patients. A further goal was to describe the feasibility of screening ED populations using the ASSIST given the inherent limitations on studying such patients contemporaneously.

## METHODS

This observational, cross-sectional study was nested within a randomized controlled trial registered at ClinicalTrials.gov (NCT01326169).^12^ Trained research assistants (RAs) screened patients in two EDs in a northeastern U.S. city. One was a Level I trauma center with 105,000 patient visits/year, of which 29% are admitted, with an average patient age of 52, 11% Hispanic/Latino, and 75% white, 14% black, and 11% other race; and the second was an academic community hospital with 55,000 patient visits/year, of which 28% are admitted, with an average patient age of 46, 20% Hispanic/Latino, and 65% white, 16% black, and 19% other race. During shifts that involved all days and times, RAs approached patients following a predetermined, randomly ordered list of treatment rooms. More recruitment shifts occurred at the Level I trauma center (61%), weekends were oversampled due to high patient volume (32% of shifts), and few shifts were scheduled between 11:30 p.m. and 7:30 a.m. (1%) due to difficulty staffing them. The RAs screened the patients’ medical records to identify patients eligible for verbal consent for additional screening. Inclusion criteria were presenting to the ED for an injury, age ≥ 18 years, English-speaking, medically stable, not admitted to the hospital, and not combative, intoxicated, or in police custody. Additional eligibility criteria pertinent to the trial were ascertained: confirmation that they identified as injured, not homeless, and had access to a telephone. Eligible participants completed the ASSIST on a tablet computer. The ASSIST has been adapted for administration via tablet computer in a prior ED-based study.^13^ The current analysis includes all participants who completed the ASSIST as part of screening for the parent trial.

For a convenience sample of 15 day and evening recruiting shifts a second RA partnered with the screening RA to record the length of time for screening with the ASSIST. The institutional review board for both hospitals approved the study and patients received no financial incentives for completing the ASSIST. Our reporting of the conduct, data analysis and interpretation of the results of this cross-sectional study is consistent with the “Strengthening the Report of Observational Studies in Epidemiology” statement.^14^

### Measures

The ASSIST has been found to have acceptable validity for assessing psychoactive substance use.^10^ A current specific substance involvement score is calculated for each substance by summing responses to six questions about prior three-month use, psychological dependence, harmful use, and lifetime and recent problems related to its use. Responses of “don’t know” or “refuse to answer” were given a value of 0. For all substances but alcohol, a score of 4 – 26 indicates moderate-risk use/abuse and an associated recommendation for a brief intervention; for alcohol, the corresponding range is a score of 11 – 26. For all substances, a score of 27 – 39 indicates high-risk use/dependence and an associated recommendation of a more intensive treatment intervention.^10^ A final question asks if injection drugs have been used; a positive response indicates high-risk substance use. Time data was collected using a stopwatch and recorded.

### Statistical Analyses

We calculated the mean and standard deviation for each substance’s specific involvement score using SAS 9.3 (Cary, NC). The mean, standard deviation, minimum, and maximum were calculated for each component of the time analysis using Microsoft Excel (Redmond, WA).

## RESULTS

Between July 2010 and March 2013, 9,788 patients were approached for screening; 5,695 completed the ASSIST. Reasons for not completing the ASSIST (see Supplemental Figure) were not meeting eligibility criteria (n=2,405) or refusing consent (n=1,688). More patients (72%) were approached for screening at the Level I trauma center than the academic community hospital, reflecting the greater volume of patients seen at the Level I trauma center. Two participants had insufficient data to calculate an ASSIST score for any substance. Substance use was common in this population, with only 434 (8%) reporting no lifetime use of any psychoactive substance (Table). Overall, 51% of participants reported moderate- or high-risk use of at least one substance. Among patients reporting risky substance use, 80% were indicated for brief intervention and 20% for more intensive treatment. Findings differed slightly by site; fewer participants at the Level I trauma center reported low-risk use (48% vs 51% at the community hospital) and more reported moderate-risk use (42% vs 40% at the community hospital) or high-risk use (10% vs 9% at the community hospital) (p<0.01).

Time data for screening was collected for 191 participants (see Supplemental Table). The average time to complete the ASSIST was 5.4 minutes (standard deviation 4.0 minutes). Of the participants who completed the ASSIST, 13 (18.6%) had to pause completing the ASSIST to allow for their clinical care to proceed. The average time of the pause for these 13 patients was 26.4 minutes (standard deviation 35.6 minutes), with a minimum of one minute and a maximum of 115 minutes.

## DISCUSSION

Alcohol and other substance use has consistently been documented among injured ED patients.^8^,^9^,^15^,^16^ Findings from this study using the ASSIST indicate that not only is substance use common among injured ED patients, but half (51%) of all patients receive ASSIST scores indicating the need for a treatment intervention. Blow et al. (2011) screened 14,557 adults presenting to an urban ED with medical complaints or injuries using the Substance Abuse Outcomes Module (SAOM) and found that 34% of patients reported risky substance use. Among injured patients, 38% needed intervention or treatment, demonstrating more risky substance use among injured patients. Hankin et al. (2013) screened 19,055 urban ED patients with either medical complaints or injuries using a modified version of the ASSIST and found that 28% of patients reported binge alcohol use or other drug use and, of those, 56% (15% of all patients screened) reported risky substance use. This is a much lower prevalence than found in the current analysis or by Blow et al. (2011). However, they modified screening by only administering the ASSIST to participants reporting prior 12-month use of tobacco, illicit drugs, or binge alcohol use and asking about the prior 30 days rather than the prior three months, both of which could have resulted in false negatives.^8^ Thus, their study is not representative of the ASSIST as a comprehensive substance-use screening tool in the ED as it was designed. We used the ASSIST as designed and validated by the WHO in a similar ED setting and found a much higher prevalence of risky substance use indicating the need for a treatment intervention, more similar to findings based on screening with the SAOM. These findings highlight the prevalence of substance misuse in an ED injured population and the importance of screening for, developing and offering substance misuse treatment resources to ED patients.

Finally, the time data for the administration of the ASSIST in the ED is very encouraging. Participants completed the ASSIST on the low end of the expected range of 5–10 minutes,^17^ despite being administered in a busy clinical setting. Less than one-fifth of patients paused their screening due to clinical care and even in the case of a long pause, as might happen when a patient needs imaging or other ED medical intervention, the patient was able to resume and complete the ASSIST. This demonstrates that the ASSIST may be a useful tool for both research and clinical programs conducting screening for risky substance use in the ED.

## LIMITATIONS

Limitations of the current study include the refusal rate (23% of those eligible) and the lack of other substance use measures to facilitate comparison to other studies. In addition, very few shifts covered overnight hours between 11:30 pm and 8:00 am. Also, patients who were critically injured or intoxicated for the duration of the RA’s shift could not be screened and patients reporting homelessness or lack of access to a telephone did not complete the ASSIST. Overall, nearly 22% of patients could not be screened due to not meeting study criteria and many of them were likely risky substance users such as those who were intoxicated. Findings are based on self-report and so may be subject to recall and social desirability biases. These all might suggest that the true prevalence of risky substance use is higher than previously estimated in this population. Finally, the ASSIST was completed on tablet computers, which may have positively impacted the completion time and ability to pause for interruptions but may not be available in all EDs.

Strengths of this study include the completion of the ASSIST as developed by the WHO by all injured patients who provided consent. It also included both a Level I trauma center and a smaller academic community hospital, demonstrating that trauma centers may have a slightly higher prevalence of risky substance use but both locations see a large volume of patients who may be indicated for a treatment intervention.

## CONCLUSION

Our findings show that the rate of substance use among injured ED patients is high and screening for substance use in the ED with the ASSIST is feasible and produces similar, albeit somewhat higher, results compared to other screening tools. This study demonstrates the feasibility of using the ASSIST in the ED setting, which may allow EDs to collect local substance-use data for multiple substances that could help determine what community referral resources and hospital-based programs are needed. The higher proportions of risky substance use found in this study may be due to differences in injured patients, i.e., that substance use is more heavily implicated in injured versus non-injured ED patient populations.

## Supplementary Information





## Figures and Tables

**Table:** Distribution of risky substance use among injured patients screened in the emergency department (N=5,695).

Substance	High-risk use	Moderate-risk use	Low-risk use	No lifetime use
Tobacco	6.17 (5.54,6.79)	34.28 (33.04,35.51)	25.37 (24.23,26.50)	34.19 (32.96,35.42)
Alcohol	3.48 (3.01,3.96)	14.32 (13.41,15.23)	70.21 (69.03,71.40)	11.98 (11.14,12.83)
Cannabis	2.21 (1.83,2.59)	19.29 (18.27,20.32)	31.08 (29.87,32.28)	47.42 (46.12,48.72)
Cocaine	0.78 (0.55,1.00)	3.23 (2.77,3.69)	14.13 (13.23,15.04)	81.86 (80.86,82.87)
Opioids	0.60 (0.40,0.80)	2.25 (1.87,2.64)	4.25 (3.72,4.77)	92.90 (92.23,93.57)
Amphetamines	0.28 (0.14,0.42)	2.61 (2.19,3.02)	10.17 (9.38,10.95)	86.94 (86.07,87.82)
Hallucinogens	0.09 (0.01,0.17)	1.55 (1.23,1.88)	12.55 (11.69,13.41)	85.81 (84.9,86.72)
Sedatives	0.06 (0,0.12)	1.03 (0.76,1.30)	3.18 (2.71,3.66)	95.73 (95.19,96.28)
Inhalants	0	0.32 (0.17,0.46)	2.59 (2.18,3.00)	97.09 (96.66,97.53)

All values are % (95% confidence interval).
