# Supplementary figures and images for: Cross-Sectional Study of Risky Substance Use by Injured Emergency Department Patients
Source: West J Emerg Med. 2017 Mar 13;18(3):345–8. doi: 10.5811/westjem.2017.1.32180 (PMC5391882; doi:10.5811/westjem.2017.1.32180)

**Supplemental Figure.** Patient screening diagram.

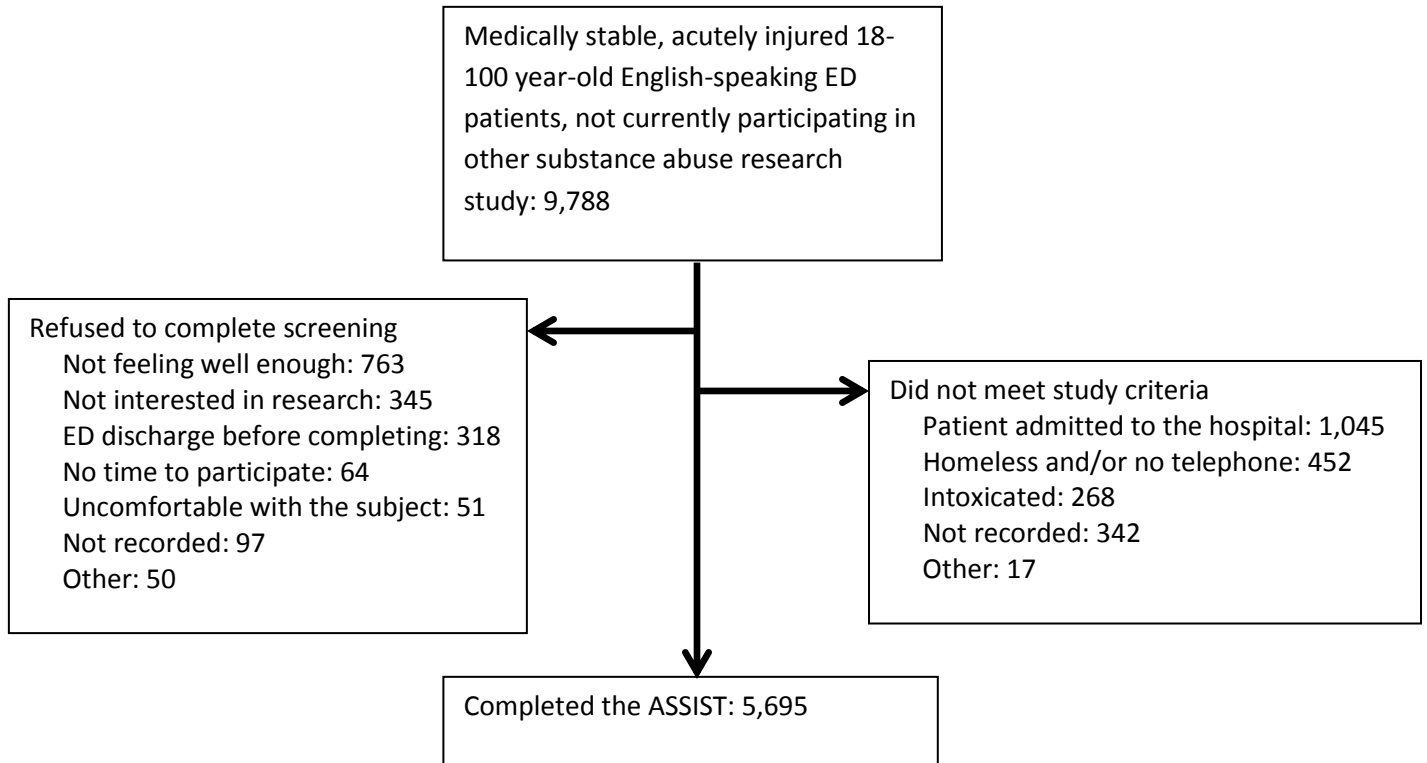

Supplement: Supplementary file 1 [file wjem-18-345-s001.pdf]
