# Supplementary material for: Cross-Sectional Study of Risky Substance Use by Injured Emergency Department Patients
Source: West J Emerg Med. 2017 Mar 13;18(3):345–8. doi: 10.5811/westjem.2017.1.32180 (PMC5391882; doi:10.5811/westjem.2017.1.32180)
Supplement: Supplementary file 2 [file wjem-18-345-s002.pdf]

Supplemental Table. Time to complete screening activities for injured patients in the emergency department.

Activity	n	Mean (Standard Deviation)	Minimum	Maximum
Reviewing randomly ordered rooms to identify potentially injured patients	191	9.1 (9.3)	0.7	44.0
Reviewing medical record for preliminary eligibility criteria	191	1.1 (0.4)	1.0	5.0
Asking participant for verbal consent, followed by screening questions if patient agrees	111	1.9 (1.3)	1.0	9.0
Completing the ASSIST	70	5.4 (4.0)	2.0	21.0

All time values measured in minutes.
ASSIST: Alcohol, Smoking and Substance Involvement Screening Test.
